# EHMTI-0114. Vestibular migraine, prevalence in vestibular and headache centre and aids for diagnosis

**DOI:** 10.1186/1129-2377-15-S1-B23

**Published:** 2014-09-18

**Authors:** C Mostardini, G Nola, R Giovanni

**Affiliations:** 1 Neurology Department, Giovan Battista Grassi Hospital, Roma, Italy; 2 Otolaringology Department, Giovan Battista Grassi Hospital, Roma, Italy; 3 Sapienza, University of Rome Dept of Otolaryngology, Italy

## Introduction

In population-based studies, lifetime prevalence of migraine and vertigo in the general population of Western industrial nations is approximately 16% and 7%, respectively. A lot terms are used to describe combination of migraine and vestibular symptoms (migranous vertigo, migraine-associated-vertigo, vertiginous migraine), recently the International Headache Society and the Barany Society (International Society for NeuroOtology) created a consensus document with diagnostic criteria for Vestibular Migraine (VM) to clearly diagnose and compare patient populations in a standardized manner.

Evaluate the prevalence of VM is particularly complex for its overlap between otolaryngology and headache experts. Our experience teaches us that, if main symptom of VM is headache, probably patient will be more easily diagnosed migraine but not VM, and if the main symptom is dizziness may not be interviewed on association with headache, failing the diagnosis.

Aim of the study is to start a collaboration with the otolaringology to evaluate the utility of self-administered tests to facilitate diagnosis of VM in patients who access to vestibular or headaches clinics.

## Methods

Patients who access from 15 April to 30 May in our clinics will fill out self-administered tests for the diagnosis of migraine as ID-Migraine, disability scales for headache and dizziness as the Headache Impact Test 6 (HIT-6) and the Italian Dizziness Handicap Inventory (DHI-I).

## Results

Preliminary data show that administration of tests has sensibly improved (more than 70%) diagnosis of VM in vestibular and in headache clinic improving sensibility of clinicians to both symptoms, optimizing diagnosis and treatment of vertigo undefined.

No conflict of interest

